# Influence of culture media, pH and temperature on growth and bacteriocin production of bacteriocinogenic lactic acid bacteria

**DOI:** 10.1186/s13568-018-0536-0

**Published:** 2018-01-24

**Authors:** En Yang, Lihua Fan, Jinping Yan, Yueming Jiang, Craig Doucette, Sherry Fillmore, Bradley Walker

**Affiliations:** 1 Faculty of Life Science and Technology, Kunming University of Science and Technology, Kunming, China; 2 Agriculture and Agri-Food Canada, Kentville Research and Development Centre, 32 Main Street, Kentville, NS B4N 1J5 Canada; 3 South China Botanical Garden, Chinese Academy of Sciences, Guang Zhou, China

**Keywords:** Lactic acid bacteria (LAB), Bacteriocins, Bacteriocin activity, Bacterial growth curves

## Abstract

There has been continued interest in bacteriocins research from an applied perspective as bacteriocins have potential to be used as natural preservative. Four bacteriocinogenic lactic acid bacteria (LAB) strains of *Lactobacillus curvatus* (Arla-10), *Enterococcus faecium* (JFR-1), *Lactobacillus paracasei* subsp. *paracasei* (JFR-5) and *Streptococcus thermophilus* (TSB-8) were previously isolated and identified in our lab. The objective of this study was to determine the optimal growth conditions for both LAB growth and bacteriocins production. In this study, various growth conditions including culture media (MRS and BHI), initial pH of culture media (4.5, 5.5, 6.2, 7.4 and 8.5), and incubation temperatures (20, 37 and 44 °C) were investigated for LAB growth measured as optical density (OD), bacteriocin activity determined as arbitrary unit and viability of LAB expressed as log CFU ml^−1^. Growth curves of the bacteriocinogenic LAB were generated using a Bioscreen C. Our results indicated that Arla-10, JFR-1, and JFR-5 strains grew well on both MRS and BHI media at growth temperature tested whereas TSB-8 strain, unable to grow at 20 °C. LAB growth was significantly affected by the initial pH of culture media (*p* < 0.001) and the optimal pH was found ranging from 6.2 to 8.5. Bacteriocin activity was significantly different in MRS versus BHI (*p* < 0.001), and the optimal condition for LAB to produce bacteriocins was determined in MRS broth, pH 6.2 at 37 °C. This study provides useful information on potential application of bacteriocinogenic LAB in food fermentation processes.

## Introduction

Lactic acid bacteria (LAB) are a diverse group of gram positive, catalase negative, oxidase negative, and micro-aerophilic organisms (Carr et al. 2002). They are generally recognized as safe bacteria (GRAS status) and play an important role in food and feed fermentation and preservation, either as the natural microflora or as starter cultures under controlled conditions (Daeschel 1989). According to Magnusson (2003), the antimicrobial effects of LAB contribute to the yield of organic acids, competition for nutrients and production of antagonistic compounds. Bacteriocins are a group of antimicrobial compounds, which are ribosomally synthesized peptides produced by bacteria to inhibit the growth of similar or closely related bacterial strains either in the same species, or across genera (Bowdish et al. 2005; Cotter et al. 2005). LAB bacteriocins have potential biotechnological applications because they are easy to produce, non-toxic to humans, stable at low pH values, and sensitive to proteases (Todorov 2009). Many bacteriocins are heat-stable and retain their activity after several months of frozen or refrigerated storage and after drying. For instance, nisin is already used as food preservative, and pediocins produced by LAB have bactericidal activity against gram-positive bacteria and are also suitable candidates for use as food bio-preservatives (Bari et al. 2005). Usually bacteriocins are produced in complex culture media including MRS, TGE, APT, BHI, TSB and TSBYE (Guerra et al. 2001; Balciunas et al. 2016; Renye et al. 2016). However, bacteriocin production can be influenced by culture conditions, such as incubation atmosphere, pH, temperature and microbial growth phase (Gänzle et al. 1999; Todorov et al. 2012; Zhang et al. 2012; Zhou et al. 2015; Turgis et al. 2016). Several studies have shown that bacteriocin production is dependent on the biomass (De Vuyst et al. 1996; Mataragas et al. 2003, 2004; Todorov and Dicks 2009; Todorov et al. 2010). However, Mataragas et al. (2003) reported that the highest bacteriocin production is determined at temperatures and pH lower than that of the optimal cell growth. Because high bacteriocin production may or may not occur at optimal growth conditions and high cell mass (Todorov and Dicks 2009; Masuda et al. 2016), this necessitates study on the relationship between bacterial growth conditions and bacteriocin production (Leroy and De Vuyst 2002).

Microbial growth curves and mathematical models can provide useful information to understand microorganism’s growth behavior and select the optimal growth conditions. Turbidimetric method is a good alternative used to study bacterial growth since optical density (OD) measurement gives a real time values of bacterial population and have practical significance when dealing with bacteria samples in high cell densities (Carlos et al. 2009; Dalgaard and Koutsoumanis 2001). Estimation of microbial growth parameters based on absorbance measurements has the advantages of being rapid, non-destructive, inexpensive and relatively easy to automate as compared to the other techniques such as classical viable counts methods (Dalgaard and Koutsoumanis 2001). In present research, turbidimetric method with Bioscreen C was used to determine how different growth conditions affected LAB growth and bacteriocin production. Four bacteriocinogenic LAB strains, Arla-10 (GenBank accession number JX275803), JFR-1 (GenBank accession number JX275814), JFR-5 (GenBank accession number JX275817) and TSB-8 (GenBank accession number JX275827), isolated from cheeses and yogurt products with anti-listerial effect were selected (Yang et al. 2012). The influences of culture media, initial pH of culture media and temperature on LAB growth and bacteriocins production were investigated, and the relationship between growth kinetics and bacteriocins production were determined.

## Materials and methods

### Culture media for bacteriocinogenic lactic acid bacteria and *Listeria innocua*

Four bacteriocinogenic LAB strains of *L. curvatus* (Arla-10), *E. faecium* (JFR-1), *L. paracasei* subsp. *paracasei* (JFR-5) and *S*. *thermophilu*s (TSB-8) were originally isolated from cheeses and yogurts in our lab. In brief, bacteriocinogenic LAB were isolated using MRS and M17 media. The agar diffusion bioassay was used to screen for bacteriocin or bacteriocin-like substances (BLS) producing LAB using *Lactobacillus sakei* and *Listeria innocua* as indicator organisms as described by Yang et al. (2012). Arla-10, JFR-1 and JFR-5 strains were cultured in MRS broth (10 g peptone, 8.0 g lab-lemco’ powder, 4.0 g yeast extract, 20 g glucose, 1 ml Tween’ 80, 2.0 g di-potassium hydrogen phosphate, 5.0 g sodium acetate 3 H_2_O, 2.0 g tri-ammonium citrate, 0.2 g magnesium sulphate 7 H_2_O, and 0.05 g manganese sulphate 4 H_2_O per liter of water, pH 6.2 ± 0.2) (Oxoid, Basingstoke, UK). TSB-8 strain was cultured in M17 broth (5.0 g tryptone, 5.0 g soya peptone, 5.0 g meat digest, 19.0 g di-dodium-β-glycerophosphate and 0.25 g magnesium sulphate per liter of water, 50 ml 10% (w/v) lactose, pH 6.9 ± 0.2) (Oxoid, UK). *Li. innocua* used as an indicator organism for the bacteriocins assay was cultured in Brain Heart Infusion broth (BHI, 7.7 g calf brain, infusion from 200, 9.8 g beef heat, infusion from 250, 10 g proteose peptone, 2.0 g dextrose, 5.0 g sodium chloride, and 2.5 g disodium phosphate per liter of water, pH 7.4 ± 0.2) (Fisher Scientific, ON, Canada). The bacteriocinogenic LAB strains were stored at − 80 °C in their culture broth adding 30% sterile glycerol (Goldman 1990).

### Preparation of inoculums

One ml of frozen LAB (*Enterococcus* sp. or *Lactobacillus* sp.) was cultured in 20 ml MRS broth, pH 6.2, while *Streptococcus* sp. was cultured in M17 broth, pH 6.9, respectively, at 37 °C for 24 h. One ml of LAB culture was then sub-cultured in MRS broth overnight and cells were harvested using a centrifuging at 14,000*g* for 5 min (Sorvall RC6 PLUS, Thermo-electron Corporation, Asheville, NC, USA). The cell pellet was washed with saline solution (0.85% NaCl) and re-suspended using 0.85% NaCl to a final optical density (O.D.) of 0.4 at 600 nm measured with a spectrophotometer (Ultrospec 3100 Pro, Biochrom Ltd. England). The cell suspensions were used as the inoculum for the growth curve experiments. *Li. innocua* was grown in BHI broth at 37 °C for 16 h, and then centrifuged at 14,000*g* for 5 min to collect bacterial cells. A final density of *Li. innocua* at 5 × 10^5^ CFU ml^−1^ was prepared using 0.1% (w/v) peptone water for bacteriocin assay study.

### Combinations of growth condition for LAB growth curve study

Two culture media of MRS and BHI broths with initial pHs adjusted to 4.5, 5.5, 6.2, 7.4 and 8.5, respectively, were prepared with 0.5 N HCl or NaOH. Three different incubation temperatures at 20, 37 and 44 °C were set up for incubation of the bacteriocinogenic LAB strains.

The growth curves for the four bacteriocinogenic LAB in MRS and BHI broth at different initial pH and temperatures were obtained with the Bioscreen C^®^ (OY Growth Curves Ab Ltd, Finland). Bioscreen C was able to measure bacterial growth kinetically and generate growth curves based on turbidity changes of samples. In brief, 15 μl of LAB cell suspensions (OD_600 nm_ = 0.4) was inoculated into 285 μl (5% level (v/v) inoculum) of each treatment combination (culture medium and pH) in Bioscreen C multi-well plates. At 37 or 44 °C, the multi-well plates were incubated for 48 h, while at 20 °C, the plates was incubated for 72 h. OD values were measured every 20 min under brown filter with a wavelength of 600 nm. All assays were performed for four times, and the data was averaged. For statistical analysis, logistic model was used and latency times, slopes, and maximum OD values were calculated.

### Relationships between LAB growth kinetics and bacteriocins production

Eight combinations of growth condition including MRS, pH 6.2 at 37 and 44 °C, MRS, pH 7.4 at 37 and 44 °C, BHI, pH 5.5 at 37 and 44 °C, and BHI, pH 6.2 at 37 and 44 °C were prepared for further investigation the impact of growth condition on LAB growth and bacteriocins activity.

0.5 ml of LAB cell suspensions (OD_600_ = 0.4) was inoculated to 9.5 ml of culture broth (5% level (v/v) inoculum) of each combination described above, respectively. At 37 °C, samples were taken at 6, 8, 10, 13, 16 and 24 h, while at 44 °C samples were taken at 5, 7, 9, 11, 13 and 18 h for determining LAB counts and bacteriocins activity (AU).

### Measurement of pH, OD values and viability of LAB (CFU ml^−1^)

The pH and OD values of LAB samples were measured following the methods described by Yang et al. (2012). For determination of viability of LAB, a series of dilution were prepared with peptone water (0.1% peptone). MRS was used to culture *Enterococcus* sp. and *Lactobacillus* sp. while M17 was used for culturing *Streptococcus* sp. At each dilution, 50 µl of sample was spiral plated to their respective culture medium (agar) plates using a Whitely Automatic Spiral Plater (WASP2) (Don Whitley Scientific Limited, Shipley, England). The petri dishes were then incubated at 37 °C for 48 h and LAB colonies were counted using an aCOLyte colony counter (Synbiosis, Cambridge, England).

### LAB bacteriocin activity (AU) test

The agar diffusion bioassay (Herreros et al. 2005) with modification by Yang et al. (2012) was used to determine LAB bacteriocin activity. At each sampling time the supernatant of LAB cultures was centrifuged at 14,000*g* for 5 min and LAB cells were moved. The cell free supernatants (CFS) were filtered through 0.22 µm syringe filters (Chromatographic Specialties Inc., ON, Canada) and then adjusted to pH 6.0 by sterilized 1 mol l^−1^ NaOH or 1 mol l^−1^ HCl to rule out inhibition effect resulting from organic acid. The neutralized supernatant was mixed with 1 mg ml^−1^ of catalase (Sigma-Aldrich Corporation, USA) at 25 °C for 30 min to eliminate the inhibitory effect of hydrogen peroxide. pH adjusted and H_2_O_2_ eliminated supernatants were filtered again to obtain bacteriocin like substance (BLS). 35 μl of untreated cell free supernatants (control) or BLS was added to the 5 mm wells in BHI agar plates (semi-solid with 0.7% agar) which contained *Li. innocua* at 5 × 10^5^ CFU ml^−1^. The plates were incubated at 37 °C for 24 h to determine if there was any inhibitory zone. In this study, the agar diffusion bioassay was again used to measure bacteriocin activity with *Li innocua* as an indicator. To quantify the bacteriocin activity, CFS or BLS was serially diluted twofold with sterile deionized water. The bacteriocins’ antibacterial activity (AU) was defined as 2^n^ × 1000 µl/35 μl, where n is the reciprocal of the highest dilution of CFS or BLS with inhibition of *Li. innocua*. The detailed procedures to determine the bacteriocin activity were described by Yang et al. (2012).

### Statistical analysis

For growth curve test, a split–split plot design was performed with the four bacteriocinogenic LAB strains on the main plot while the two culture media and five initial pH values on the sub plot, which was split into three different temperatures. Bioscreen C with 10 × 10 layout multiwell plates was used and growth curves data were analyzed from fitted data of a logistic model. The logistic template formula is expressed as Y = A + C/(1 + EXP (− B * (t − M)), where A = the fitted initial level; B = the relative growth rate; A + C = the final population density; t = time points; M = the inflection point.

For bacteriocin production assay, analysis of growth curves was done from fitted data of spline model. Spline template equation is expressed as Y = a + bS (x; 2), where a = the intercept; b = the slope; S = the spline function; x = the measured values. All experiments were repeated four times.

## Results

### Different culture conditions affected LAB growth

For this study, different combinations of growth condition were used to culture four bacteriocinogenic LAB strains. The growth curves were generated by Bioscreen C. As shown in Table 1, growth conditions affected latency times, slopes, and maximum OD values of LAB. All bacteriocinogenic LAB were able to grow at 20, 37 and 44 °C, except for TSB-8, which was unable to grow at 20 °C. Culture media, initial pH and growth temperature significantly affected the latency time of the four LAB strains (*p* ≤ 0.001, Table 2). Comparing two media used, the latency time for all LAB strains was shorter in BHI broth than in MRS broth. When using the same culture medium, the higher growth temperature resulted in the shorter latency time. LAB growth measured as OD value was significantly affected by the pH of medium (*p* < 0.001, Table 2). Higher OD_600 nm_ values were recorded in the medium with initial pHs between 6.2 and 8.5. In contrast, the four LAB strains were unable to grow at pH 4.5 regardless of media or incubation temperatures chosen (Table 1).

**Table 1:** Growth kinetics of the four bacteriocinogenic LAB strains under different growth conditions

Isolates	Arla-10			JFR-1			JFR-5			TSB-8		
Culture conditions	Slope^a^	Latency time (h)	A_max_^b^	Slope	Latency time (h)	A_max_	Slope	Latency time (h)	A_max_	Slope	Latency time (h)	A_max_
20 °C, MRS pH 5.5	1.37	25.30	0.63	1.17	25.01	0.58	0.87	22.21	0.79	0.05	5.41	0.16
20 °C, MRS pH 6.2	0.97	11.66	1.04	0.77	11.74	0.89	0.77	16.41	0.88	0.01	4.31	0.17
20 °C, MRS pH 7.4	1.05	10.28	1.22	1.08	11.29	1.22	0.59	25.27	0.71	0.06	4.00	0.19
20 °C, MRS pH 8.5	0.96	11.66	1.15	0.91	12.01	1.12	0.56	21.76	0.68	0.01	5.97	0.24
20 °C, BHI pH 5.5	0.54	8.33	0.62	0.58	8.61	0.66	0.44	14.10	0.46	0.09	19.72	0.16
20 °C, BHI pH 6.2	0.80	6.53	0.94	0.97	5.79	0.99	0.59	13.02	0.62	0.13	21.88	0.16
20 °C, BHI pH 7.4	0.82	8.01	0.92	0.84	7.91	0.93	0.54	15.09	0.55	0.51	38.52	0.23
20 °C, BHI pH 8.5	0.78	8.94	0.88	0.80	9.08	0.90	0.42	14.78	0.47	0.62	43.25	0.24
37 °C, MRS pH 5.5	0.56	3.85	0.67	0.56	3.81	0.67	0.42	7.83	0.45	0.32	2.96	0.43
37 °C, MRS pH 6.2	0.92	3.07	1.03	0.92	3.11	1.03	0.68	7.39	0.79	0.44	5.50	0.58
37 °C, MRS pH 7.4	0.95	3.00	1.10	0.91	3.01	1.06	0.83	7.50	1.00	0.67	4.30	0.65
37 °C, MRS pH 8.5	0.92	3.74	1.15	0.93	4.10	1.15	0.77	8.56	1.00	0.47	5.63	0.68
37 °C, BHI pH 5.5	0.70	2.73	0.79	0.75	2.47	0.82	0.62	5.39	0.71	0.44	5.69	0.48
37 °C, BHI pH 6.2	0.93	2.39	1.02	0.92	2.35	1.01	0.81	4.70	0.93	0.62	6.30	0.70
37 °C, BHI pH 7.4	0.99	2.63	1.09	1.00	2.66	1.09	0.94	5.19	1.05	0.67	9.57	0.68
37 °C, BHI pH 8.5	0.95	2.85	1.06	0.94	2.85	1.04	0.89	5.55	0.99	0.72	4.16	0.82
44 °C, MRS pH 5.5	0.05	2.37	0.16	0.27	2.31	0.39	0.10	2.70	0.24	0.40	3.96	0.41
44 °C, MRS pH 6.2	0.31	2.53	0.45	0.52	2.13	0.66	0.23	3.07	0.38	0.50	3.15	0.36
44 °C, MRS pH 7.4	0.55	2.66	0.70	0.75	2.38	0.92	0.30	3.63	0.47	0.39	2.45	0.48
44 °C, MRS pH 8.5	0.58	3.51	0.79	0.75	3.18	0.97	0.25	6.06	0.47	0.51	2.90	0.23
44 °C, BHI pH 5.5	0.64	2.38	0.78	0.80	2.18	0.89	0.45	1.46	0.55	0.65	2.21	0.59
44 °C, BHI pH 6.2	0.62	2.33	0.71	0.85	1.90	0.93	0.43	1.47	0.51	0.48	2.33	0.56
44 °C, BHI pH 7.4	0.59	2.32	0.67	0.82	2.11	0.90	0.36	1.40	0.44	0.46	2.68	0.46
44 °C, BHI pH 8.5	0.66	2.37	0.75	0.86	2.33	0.94	0.39	1.13	0.48	0.42	4.32	0.50

^a^ The slope of growth curves

^b^ OD (the maximum absorbency under λ_600 nm_)

**Table 2:** *p* values of single or multiple factors affecting the growth of the four bacteriocinogenic LAB strains

Parameter	Arla-10			JFR-1			JFR-5			TSB-8		
	Slope^a^	Latency time	A_max_^d^	Slope	Latency time	A_max_	Slope	Latency time	A_max_	Slope	Latency time	A_max_
Temperature (T)	0.071^b^	< 0.001	*^c^	< 0.001	< 0.001	< 0.001	*	< 0.001	*	*	0.001	*
Medium (M)	< 0.001	< 0.001	< 0.001	< 0.001	< 0.001	< 0.001	0.052	< 0.001	*	< 0.001	< 0.001	< 0.001
pH	< 0.001	< 0.001	< 0.001	< 0.001	< 0.001	< 0.001	< 0.001	< 0.001	< 0.001	< 0.001	0.009	< 0.001
T × M	< 0.001	< 0.001	< 0.001	< 0.001	< 0.001	< 0.001	< 0.001	< 0.001	< 0.001	*	< 0.001	0.005
T × pH	< 0.001	< 0.001	< 0.001	< 0.001	< 0.001	*	0.002	< 0.001	0.001	*	0.002	0.043
M × pH	< 0.001	< 0.001	< 0.001	< 0.001	< 0.001	< 0.001	*	< 0.001	*	*	< 0.001	*
T × M × pH	< 0.001	< 0.001	< 0.001	< 0.001	< 0.001	< 0.001	*	< 0.001	*	0.043	0.012	*

^a^ The slope of growth curves

^b^ *p* values

^c^ Not significant (*p* > 0.05)

^d^ The OD (the maximum absorbency under λ_600 nm_)

**Fig. 1:**
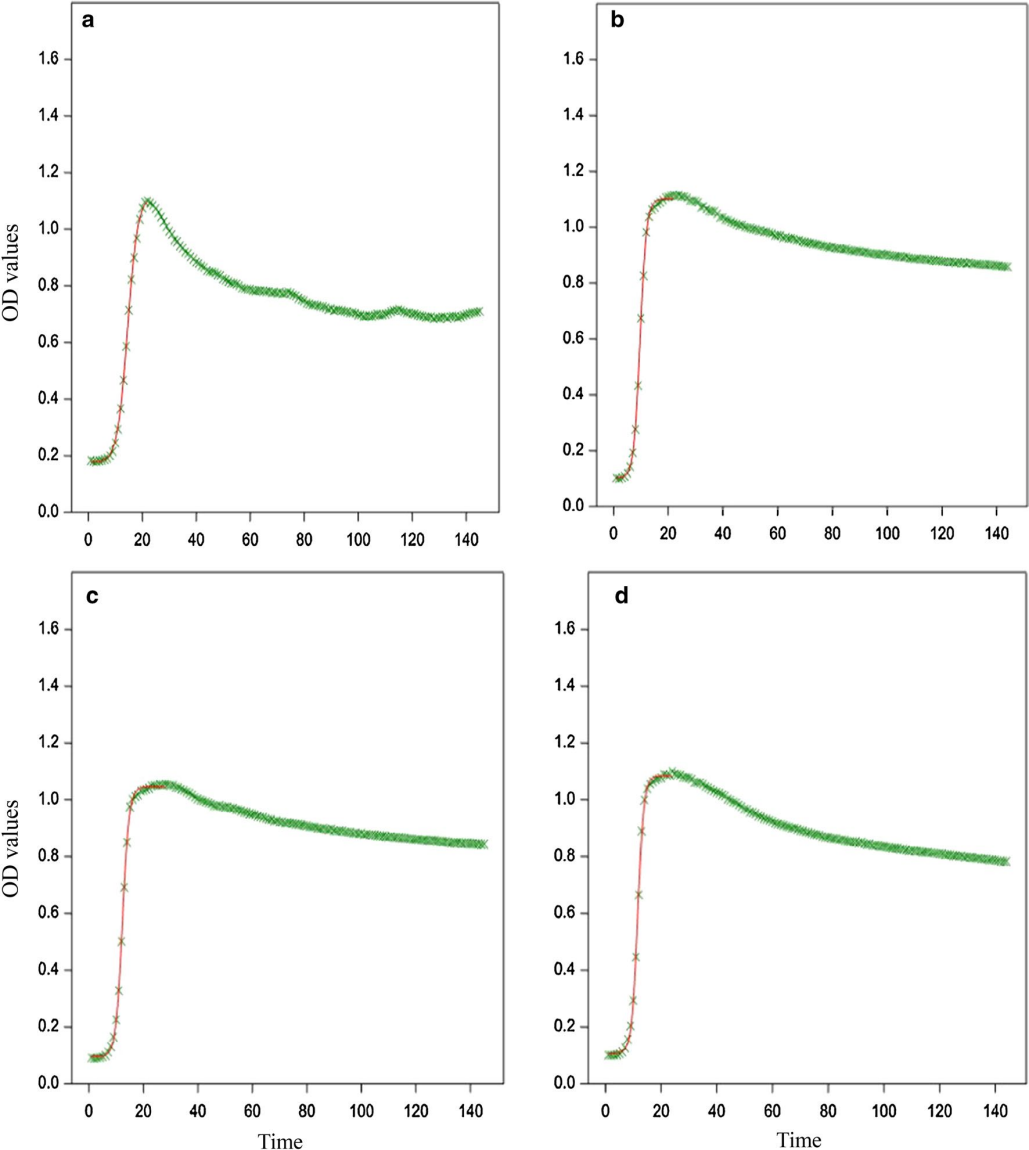
Growth curves generated from the Bioscreen C for *Lactobacillus curvatus* (Arla-10), *Enterococcus faecium* (JFR-1), *Lactobacillus paracasei* subsp. *paracasei* (JFR-5), and *Streptococcus thermophilus* (TSB-8) at their optimal growth conditions for 48 h. Arla-10 strain was cultured in MRS broth, initial pH 7.4, at 37 °C (**a**); JFR-1 strain was cultured in BHI broth, initial pH 7.4, at 37 °C (**b**); JFR-5 strain was cultured in BHI broth, initial pH 7.4, at 37 °C (**c**); TSB-8 strain was cultured in BHI broth, initial pH 8.5, at 37 °C (**d**). *The OD value was measured and recorded by the Bioscreen C at 20-min interval. The numbers of 20, 40, 60, 80 100, 120 and 140 on the X axis represent that LAB has grown for 400, 800, 1200, 1600, 2000, 2400 and 2800 min, respectively

Based on statistical analysis of growth curve parameters including latency time, slope generated by the logistic model, and maximum OD_600 nm_ value, it was concluded that the best growth condition for Arla-10 was in MRS broth with initial pH 7.4 and incubation at 37 °C (Fig. 1a), JFR-1 and JFR-5 were in BHI broth with initial pH 7.4 and incubation at 37 °C (Fig. 1b, c) while TSB-8 was in BHI broth with initial pH 8.5 and incubation at 37 °C (Fig. 1d).

### Influence of culture media, initial pH and growth temperature on LAB growth and bacteriocin production

Based on the growth kinetics results (Table 1), eight growth conditions including MRS, pH 6.2 at 37 °C, MRS, pH 6.2 at 44 °C, MRS, pH 7.4 at 37 °C, MRS, pH 7.4 at 44 °C, BHI, pH 5.5 at 37 °C, BHI, pH 5.5 at 44 °C, BHI, pH 6.2 at 37 °C, and BHI, pH 6.2 at 44 °C were chosen for determining optimal conditions for bacteriocin production.

**Fig. 2:**
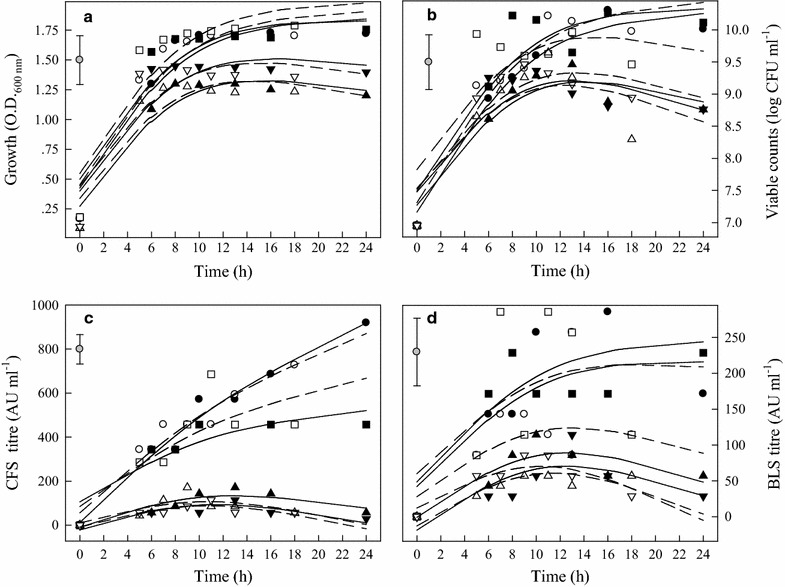
OD values, viability, and antimicrobial activities (AU) of CFS and BLS of Arla-10 strain at different growth conditions. (●) MRS, pH 6.2, 37 °C; (○) MRS, pH 6.2, 44 °C; (■) MRS, pH 7.4, 37 °C; (□) MRS, pH 7.4, 44 °C; (▲) BHI, pH 5.5, 37 °C; (△) BHI, pH 5.5, 44 °C; (▼) BHI, pH 6.2, 37 °C; (▽) BHI, pH 6.2, 44 °C. **a** Optical density (OD); **b** LAB viable counts; **c** antimicrobial activity of CFS and **d** antimicrobial activity of BLS. The experimental data were fitted to the logistic model. The vertical bar represents standard errors of the means

The effect of the eight growth combinations on OD, cell counts, bacteriocin activity of CFS and bacteriocin activity of BLS of Arla-10 strain is summarized in Fig. 2. The growth rates (OD) were higher in MRS (pH 7.4 and 6.2) than in BHI (6.2 and 5.5) (Fig. 2a, b). The highest bacteriocin activity of CFS was recorded 918.9 AU, when grew in MRS broth, pH 6.2 at 37 °C for 24 h (Fig. 2c). There was no significant difference in bacteriocin activity of CFS between 37 °C and 44 °C (Fig. 2c). However, temperature resulted in significant difference in bacteriocin activity of BLS in MRS broth, pH 6.2. The highest activity was recorded 285.7 AU in MRS, pH 6.2 at 37 °C for 16 h (Fig. 2d).

**Fig. 3:**
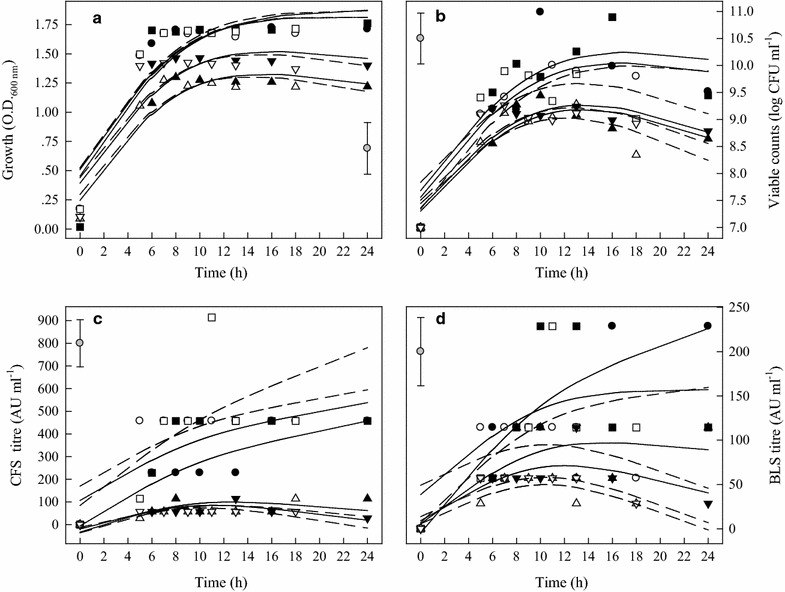
OD values, viability, and antimicrobial activities (AU) of CFS and BLS of JFR-1 strain at different growth conditions. (●) MRS, pH 6.2, 37 °C; (○) MRS, pH 6.2, 44 °C; (■) MRS, pH 7.4, 37 °C; (□) MRS, pH 7.4, 44 °C; (▲) BHI, pH 5.5, 37 °C; (△) BHI, pH 5.5, 44 °C; (▼) BHI, pH 6.2, 37 °C; (▽) BHI, pH 6.2, 44 °C. **a** Optical density (OD); **b** LAB viable counts; **c** antimicrobial activity of CFS and **d** antimicrobial activity of BLS. The experimental data were fitted to the logistic  model. The vertical bar represents standard errors of the means

For JFR-1 strain, the bacteriocin activity of CFS produced in MRS broth, pH 7.4 was higher than in MRS broth, pH 6.2. The highest bacteriocin activity of CFS was recorded in MRS broth, pH 7.4 at 44 °C for 11 h (Fig. 3c). In contrast, the highest bacteriocin activity of BLS was measured at 16 h and maintained at this level to 24 h in MRS, pH 6.2 at 37 °C (Fig. 3d).

**Fig. 4:**
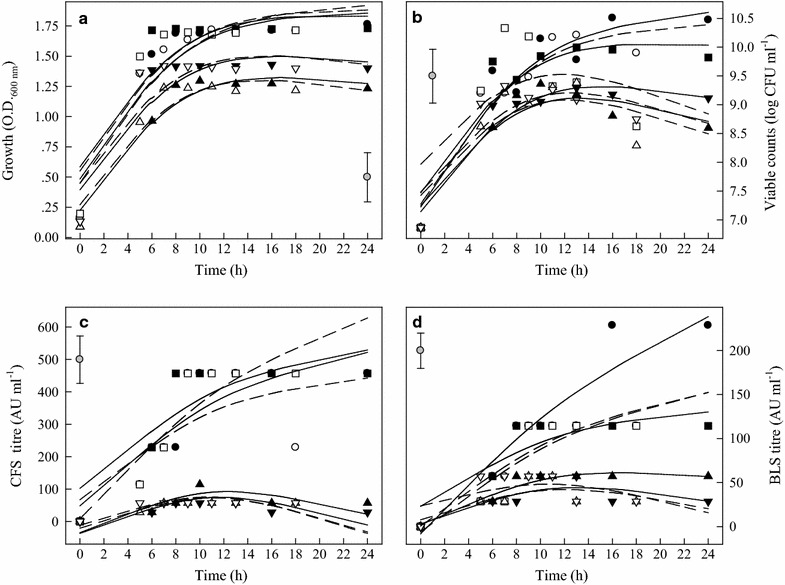
OD values, viability, and antimicrobial activities (AU) of CFS and BLS of JFR-5 strain under different growth conditions. (●) MRS, pH 6.2, 37 °C; (○) MRS, pH 6.2, 44 °C; (■) MRS, pH 7.4, 37 °C; (□) MRS, pH 7.4, 44 °C; (▲) BHI, pH 5.5, 37 °C; (△) BHI, pH 5.5, 44 °C; (▼) BHI, pH 6.2, 37 °C; (▽) BHI, pH 6.2, 44 °C. **a** Optical density (OD); **b** LAB viable counts; **c** antimicrobial activity of CFS and **d** antimicrobial activity of BLS. The experimental data were fitted to the logistic  model. The vertical bar represents standard errors of the means

For JFR-5 strain, when it was cultured in the same medium, higher initial medium pH resulted in the better growth (higher OD values) with higher viable counts (Fig. 4a, b). However, initial medium pH did not make significant difference in bacteriocin activity of CFS in MRS broth at 37 °C (Fig. 4c). The highest activity of the bacteriocin activity of BLS was determined  235.71 AU in MRS broth, pH 6.2 at 37 °C for 16 h while the viable counts of JFR-5 strain was also reached to the highest (Fig. 4b, d).

**Fig. 5:**
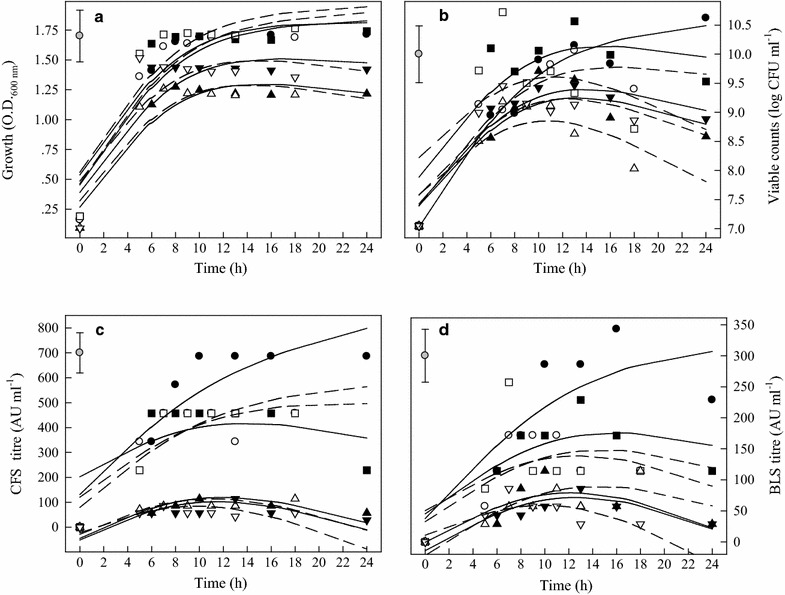
OD values, viability, and antimicrobial activities (AU) of CFS and BLS of TSB-8 strain under different growth conditions. (●) MRS, pH 6.2, 37 °C; (○) MRS, pH 6.2, 44 °C; (■) MRS, pH 7.4, 37 °C; (□) MRS, pH 7.4, 44 °C; (▲) BHI, pH 5.5, 37 °C; (△) BHI, pH 5.5, 44 °C; (▼) BHI, pH 6.2, 37 °C; (▽) BHI, pH 6.2, 44 °C. **a** Optical density (OD); **b** LAB viable counts; **c** antimicrobial activity of CFS and **d** antimicrobial activity of BLS. The experimental data were fitted to the logistic  model. The vertical bar represents standard errors of the means

When TSB-8 strain was cultured in a culture medium with higher initial pH, it reached to stationary phase faster than those cultured at lower initial pH (Fig. 5a, b). It grew well in both MRS and BHI with viable cell counts more than 10^8^ CFU ml^−1^ following incubation for 18 h, however, higher bacteriocin production was determined in MRS broth compared with that in BHI (Fig. 5c). Growth temperature also significantly affected bacteriocin activity of CFS and BLS (Fig. 5c, d). When TSB-8 strain was cultured in MRS broth, initial pH 6.2, at 37 °C for more than 10 h, CFS and BLS had the highest bacteriocin activity (AU) of 685.7 and 342.84, respectively (Fig. 5c, d).

**Fig. 6:**
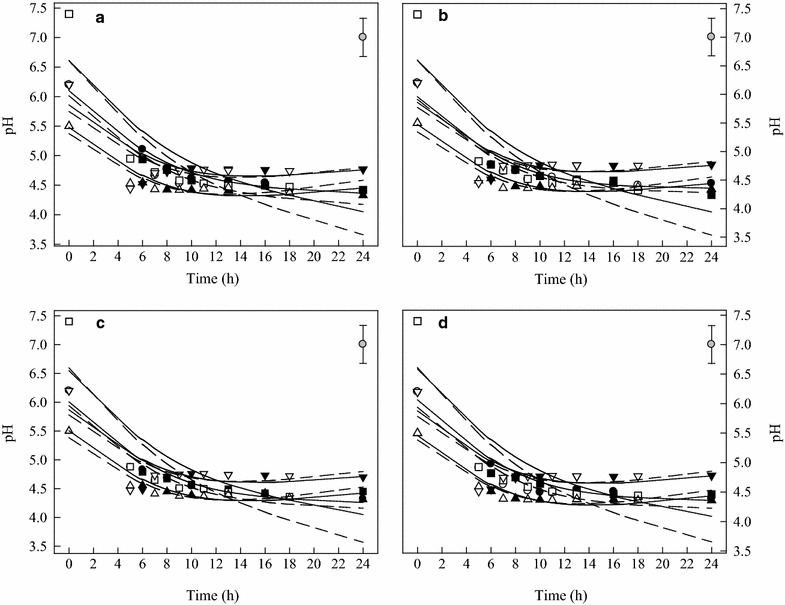
pH changes of *Lactobacillus curvatus* (Arla-10) (**a**), *Enterococcus faecium* (JFR-1) (**b**), *Lactobacillus paracasei* subsp. *paracasei* (JFR-5) (**c**), and *Streptococcus thermophilus* (TSB-8) (**d**) grown under different growth conditions. (●) MRS, pH 6.2, 37 °C; (○) MRS, pH 6.2, 44 °C; (■) MRS, pH 7.4, 37 °C; (□) MRS, pH 7.4, 44 °C; (▲) BHI, pH 5.5, 37 °C; (Δ) BHI, pH 5.5, 44 °C; (▼) BHI, pH 6.2, 37 °C; (▽) BHI, pH 6.2, 44 °C. The experimental data were fitted to the logistic  model. The vertical bar represents standard errors of the means

Over all, the results showed that culture medium and pH played an important role in bacteriocin production. It was found that following 16 h incubation, the BHI broth with initial pH of 7.4 dropped to 5.8 ± 0.2, with initial pH 6.2 dropped to 4.6 ± 0.2 (Fig. 6). Although initial pHs decreased, the bacteriocin activity was only detected in BHI broth with initial pH 6.2. On the other hand, pH was decreased dramatically when LAB grew in MRS than in BHI (Fig. 6), and higher bacteriocin production was also measured in MRS. LAB bacteriocin production was significantly different in MRS or BHI (*p* < 0.001). Four LAB strains cultured in MRS broth produced higher bacteriocins levels (AU) than that in BHI broth. The optimum conditions for bacteriocin production were determined when LAB were cultured in MRS broth at pH 6.2 and 37 °C.

### Correlations of OD value, pH, bacteriocins activity (AU) and viability of LAB

While the correlation coefficient measures a degree to which two variables are related. The strength of the relationship varies in degree based on the value of the correlation coefficient. A value of 1.0 means that there is a perfect positive relationship between the two variables while a value of − 1.0 means that there is a perfect negative relationship between the two variables. If the value is 0, this suggests that there is no relationship between the two variables. In our study, the correlation coefficient among LAB growth (OD_600 nm_), bacteriocin activity of CFS, bacteriocin activity of BLS and the viability was determined. It was founded that the decrease of initial medium pH was negatively related to LAB growth. The bacteriocin activity of CFS and BLS produced by the four LAB strains correlated with their biomass growth (r > 0.75). On the other hand, the bacteriocin activity of CFS was highly correlated with that of BLS of *L. curvatus* (Arla-10), *E. faecium* (JFR-1), *L. paracasei* subsp. *paracasei* (JFR-5) and *S. thermophilus* (TSB-8) with the r = 0.89, 0.82, 0.94 and 0.95, respectively.

## Discussion

Lactic acid bacteria are generally recognized as safe bacteria in fermented foods like dairy products, processed vegetables and play an important role in preservation. We need to better understand the factors that affect LAB growth and bacteriocin activity (production) in order to apply bacteriocinogenic LAB and/or bacteriocins in the food products. We found that initial pH level of culture medium was one of the key factors influencing the growth of the four bacteriocinogenic LAB strains. The optimal pH for the four LAB growth were pH 7.4 and 8.5 (Table 1), while they were unable to grow at a low pH of 4.5, which was supported by other researches. LeBlanc et al. (2004) reported that the growth of *La. fermentum* CRL 722 was noticeably slower at pH 4.5 (μ_max_ = 0.78 h^−1^) than at other pH values including pH 5.0, 5.5, and 6.0 (μ_max_ = 1.15 − 1.25 h^−1^). Similar results were found by Mataragas et al. (2003) that at pH 4.5, the final biomass and bacteriocin activity of *Luconostoc mesenteroides* L124 and L442 were very low, and the optimum pH for these LAB to grow were between pH 6.0 and 6.5. It was suggested that the LAB growth was suppressed at pH < 5.0.

Temperature also played an important role in LAB growth, particularly influenced the latency time (*p* ≤ 0.001, Table 2). Carlos et al. (2009) reported that *Enterococci* sp. grew in both 2YT (2× yeast tryptone medium) and skim milk at 30 and 37 °C, however, the growth curve at 30 °C presented longer lag phase compared to 37 °C. Gardini et al. (2001) found that the most important factor influencing the lag phase of *E. faecalis* was temperature, although its influence on the final cell yield was low.

Bacteriocin production is strongly dependent on medium composition (Gänzle et al. 1999). MRS medium is a rich medium with a mixture of different carbon sources and complex nitrogen sources. BHI is a general purpose nutrient medium recommended for the cultivation of a variety of microorganisms including bacteria, yeasts and mold. Growth kinetics results showed that BHI broth with initial pH 7.4 was an ideal growth condition for LAB (Table 1). However, the bacteriocin activity was not found when LAB strains were cultured in BHI broth with initial pH 7.4 at 37 °C for 16 h. This finding was consistent with the report by Khalil et al. (2009) that the highest bacteriocin inhibitory effect was obtained in MRS broth incubated at 30 °C for 12–18 h while BHI medium was not suitable for bacteriocin production. Moreover, the lower antimicrobial activity (200 AU ml^−1^) of bacteriocin ST151BR was recorded in M17 broth, BHI broth, soymilk and molasses in comparison to in MRS broth (6400 AU ml^−1^) despite relatively good growth (Todorov and Dicks 2004). Similar results were reported by De Kwaadsteniet et al. (2005) that MRS broth was the optimal medium for bacteriocin production of *E. mundtii* ST15 compared to BHI broth, M17 broth, soymilk and molasses. In our previous studies, the bacteriocin activity of LAB was not detected following incubation for 16 h in M17 medium. Therefore, some culture media used for the cultivation of microorganisms may not be suitable medium for bacteriocins production.

Some research found that the produced antimicrobial metabolites were pH-dependent, but the antifungal activity could not be attributed to a low pH effects (De Muynck et al. 2004; Elsanhoty 2008). In addition, the dependence of bacteriocin production on pH suggested that the expression of the biosynthetic genes may be regulated by pH, as has been reported previously for several classes of genes (Olson 1993). Guerra and Pastrana (2003) reported that higher pH drops, defined as the difference between initial and final pH, enhanced both nisin and pediocin production until a final pH was inappropriate for survivability and cell growth of LAB. Similar result was reported by Cabo et al. (2001), they concluded that pH drop gradient (*VpH*) enhanced nisin production (approximately fourfold), and increased the efficiency of nutrient consumption. This may be related to the need for a low final pH for an efficient post-translational processing of *L. lactis* and *P. acidilactici* to produce active bacteriocins (Yang and Ray 1994). It is also interesting to note that the buffer capacity of the different media because these bacteriocinogenic LAB strains likely acidify the medium. In addition, pH is known to have an influence on cationic peptides to associate with the cell membrane of the producer strains.

Bacteriocin production by LAB has been reported as a temperature-sensitive process (Leroy and De Vuyst 1999). In this research, bactriocins produced were strongly influenced by temperatures, especially in MRS broth, pH 6.2. Although, cultured at 44 °C resulted in a fast cell growth, which did not significantly affect final OD values and the highest bacteriocin production. It has been suggested that bacteriocin production by LAB was enhanced by sub-optimal fermentation condition (De Vuyst et al. 1996; Delgado et al. 2005). It is interesting to note that MRS broth, pH 6.2, at 37 °C provided the optimal condition for the four LAB to produce highest bacteriocin production, while the optimal pH for growth (highest OD values) was in MRS broth with pH 7.4 (Table 1). Moreover, LAB grew better in BHI broth, pH 6.2 than in BHI, pH 5.5, higher bacteriocin activity (BLS) was recorded in BHI broth, pH 5.5 (Figs. 2d, 3d, 4d, 5d). Messens et al. (2003) found that the optimum pH 6.0 for cell growth differed significantly from the optimum pH 5.1 for maximum curvacin A activity produced by *L. curvatus*. At a constant temperature of 35 °C, maximum cell yields were achieved at pH of 7.5–8.0, but this did not coincide with high enterocin production, and the highest enterocin activity of the supernatant was found at pH 5.5–6.5 (Leroy and De Vuyst 2002). Mataragas et al. (2003) also reported that the optimum pH and temperature for *Leuconostoc mesenteroides* L124 and *L. curvatu* L442 growth were 6.0–6.5 at 30 °C and for bacteriocin production was 5.5 at 25 °C.

Moreover, the bacteriocins activity of the four LAB strains was increased continuously during the exponential growth phase and the highest AU was reached by the end of this phase (Figs. 2d, 3d, 4d, 5d). The maximum bacteriocins produced in the middle or at the end of the exponential growth phase or at the beginning of the stationary phase were reported by other researchers (Guerra and Pastrana 2003; Todorov and Dicks 2004). Only a few bacteriocins were produced during the stationary phase (Lisboa et al. 2006; Khalil et al. 2009). Therefore, it is necessary to determine the correlations of pH, OD, bacteriocin activity and viability of LAB strains.

The BLS from the four bacteriocinogenic LAB were treated with 1 mg ml^−1^ of proteolytic enzymes, including proteinase K (33 U mg^−1^), α-chymotripsin (66 U mg^−1^), and trypsin (105 U mg^−1^) at 37 °C for 2 h (Yang et al. 2012). We found that the BLS lost their antilisterial activity following treatment with these enzymes indicating the proteinaceous nature of the BLS. We also investigated the effect of pH on the adsorption/desorption of BLS to LAB cells. Bacteriocins produced by JFR-1, JFR-5, Arla-10 and TSB-8 were maximally adsorbed to their cells at pH 6, 5, 5 and 4, respectively. Tricine–SDS-PAGE is the preferred electrophoretic system for the resolution of proteins smaller than 30 kDa. Using this system, we found that the molecular weights of the bacteriocins produced by these four LAB strains ranged from 4.5–6 kDa. However, it remains to be determined whether these bacteriocins will be functional in foods or feeds. Bacteriocins may be used as biopreservatives either as powdered food ingredients, purified-or partially purified-peptides or through bacteriocinogenic LAB cultures. The application of combinations of different LAB-bacteriocins may contribute to reducing possible development of resistant bacterial populations and improving the safety/quality and shelf-life of food products. Further research is required to gain insights into the molecular mechanisms involved in bacteriocin production, immunity and mode of action.

## Abbreviations

LAB: lactic acid bacteria
AU: arbitrary unit
MRS: de Man, Rogosa and Sharpe
BHI: brain heart infusion
TGE: tryptone glucose extract
APT: all-purpose with tween
TSB: trypticase soy broth
TSBYE: trypticase soy broth with yeast extract
CFU: colony forming unit
OD: optical density
GRAS: generally recognized as safe
CFS: cell free supernatants
BLS: bacteriocin like substance
